# ECHA ARN documents: chemical grouping without a toxicological rationale

**DOI:** 10.1007/s00204-023-03479-3

**Published:** 2023-03-22

**Authors:** Andreas Natsch, Greg Adamsson, Vanessa Rocha

**Affiliations:** 1 Fragrances S&T, Ingredients Research, Givaudan Schweiz AG, Kemptpark 50, CH-8310 Kemptthal, Switzerland; 2 Regulatory Affairs & Product Safety Givaudan Fragrance, East Hanover, NJ USA

## Abstract

The EU chemical strategy for sustainability (CSS) plans to use chemical grouping to “prioritise (…) substances for restrictions for all uses through grouping, instead of regulating them one by one”. Thus, toxicological grouping will become a key tool used by regulatory authorities in Europe. Over the last 2 years, ECHA has published a high number of documents labelled “Assessment of Regulatory Needs (ARN)” which are based on groups of chemicals based on structural considerations. The ARN documents are legally non-binding, yet they present the public impression of a conclusion about restrictions for groups or sub-groups of chemicals and hence may set a precedent for further binding actions. ECHA has set out definitions on what is considered a group in REACH Annex XI. However, as shown in this commentary based on five examples, the ARN do not follow these principles and propose toxicological groupings without taking into consideration mode of action and the toxicological information on the chemicals. Given the emphasis on grouping projected by the CSS, the groupings in the ARN set an unfortunate precedent on what a toxicological group means and they do not follow clear scientific standards or established toxicological principles. They also lead to a public image of guilt by association for chemicals, without any recourse for registrants to establish the scientific basis for their safe use, as presented within REACH registrations.

## Introduction

In October 2020, the European Union published the ‘Chemicals Strategy for Sustainability’ (CSS) as part of Europe’s Green Deal (European_Commission 2020). The CSS proposes grouping of chemicals as an important tool for regulatory action and substance restriction, proposing to use grouping to “*prioritise (…) substances for restrictions for all uses and through grouping, instead of regulating them one by one*”. The CSS further proposes a “*gradual move away from as-sessing and regulating chemicals substance-by-substance to regulating them by groups*” and “*favouring the assessment by groups of substances with structural or functional similarities*” (European_Commission 2020).

Hence, the concept of grouping will receive a prominent function under the CSS, yet no definition of what is meant by grouping was put forward, and the only legally binding (and toxicologically founded) definition is the one set out in REACH Annex XI, where groups are defined as follows: “*Substances whose (…) toxicological (…) properties are likely to be similar or follow a regular pattern as a result of structural similarity, may be considered as a group (…).The similarities may be based on any of the following: A common functional group; (…) common breakdown products (…) a constant pattern in the changing of the potency of the properties across the category*”.

Thus Annex XI clearly defines how toxicological groups are formed with an emphasis on *similar toxicological properties* as a result of structural similarity or a common metabolite and this definition is based on the toxicological knowledge that in specific cases molecules with similar functional reactive groups, similar overall structure or common metabolites lead to similar apical outcome in case these structural features are the basis for a shared toxicological mode-of-action.

## Assessments of groupings in ARN documents

**Fig. 1:**
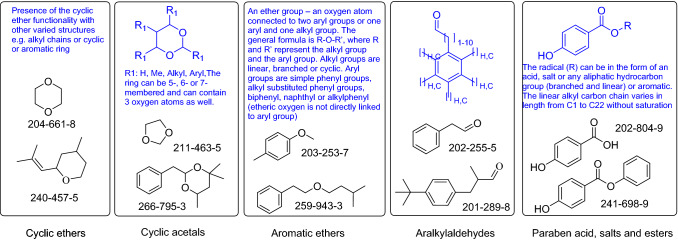
The chemical definitions made for the ARN groupings discussed in this commentary: In blue (top of each box) is the definition of the group as given in the ARN dossiers, in black (bottom of each box) two examples are given of group members specifically listed by ECHA in the dossier. Note: Some ARN group definitions are based on drawn general structures and some only based on text as shown in blue. For the cyclic ethers as an example, it is obvious that the group definition (in blue) is very vague, and that the two examples of chemicals in the group are widely different (color figure online)

In parallel to the CSS, ECHA has started to publish a large number of documents on the Assessment of Regulatory Needs (ARN) which contains a range of proposed restrictions, including potential bans of ingredients based on chemical grouping defined only based on common structural features. However, for the five exemplary cases (Fig. 1) discussed below, the grouping as defined in the ARN appear to violate the principles of Annex XI. In all five cases, there is no toxicological evidence given to justify them as groups for a toxicological assessment. In the absence of a new definition for grouping underlying the formation of the groups in the ARN, they appear arbitrary and not scientifically founded.

1. Cyclic ethers^1^

This group is defined by “*the presence of the cyclic ether functionality*”. It encompasses 35 completely different structures from a molecular weight of 72–216 Da. Currently, there is no known toxicity which is shared by ethers in general, nor is there a reason from a toxicological stand-point why a cyclic ether in principle is different from a linear ether. As key toxicological concern identified in the group is the carcinogenicity 1B (mainly to the liver) of 1,4-dioxan (EC 204-661-8) and carcinogenicity 2 for tetrahydrofuran (EC 203-726-8). An additional concern for male reprotoxicity based on a 28 d screening study was identified for tetrahydro-4-methyl-2-(2-methylprop-1-enyl)pyran (EC 240-457-5), although these effect were not reproduced in a subsequent 90 days study. Effects on female reproduction/maternal care were observed for 2-methyl-tetrahydrofuran (EC 202-507-4). The structure of these molecules of concern are widely different, and the effects observed in animal studies are not comparable and do not point to a common mode of action. At the moment, there is no reason to link toxicity neither to the presence of the ether functionality nor the fact that these are cyclic rather than linear ethers. Hence, the mere presence of a cyclic ether functionality so far would not be expected to lead to *similar toxicological properties* for the 35 extremely diverse chemicals included in the group and the requirement for grouping them are, therefore, not given.

(B) Cyclic acetals from aldehydes^2^

This group is defined by “*the presence of a cyclic acetal functionality, whereby the ring can be a 5–7-membered and contain an additional oxygen*”. In principle—based on structure—all cyclic acetals are also a subgroup of the cyclic ethers as defined in GMT 208. As for the cyclic ethers, there is currently no common toxicological property known for acetals in general nor is there any indication that cyclic acetals are different from their non-cyclic analogues. Furthermore, no reason is given why the ring should be 5–7 membered. The key toxicologial concern identified in the group is an effect of 1,3-dioxolane (EC 211-463-5) on developmental endpoints, although this effects are only observed at dosed toxic to the dam. So far, there is no indication that the observed effect is attributed the acetal functionality. In principle acetals can hydrolyse under strongly acidic conditions and 1,3-dioxolane under acidic conditions can release formaldehyde (Andrews 1987). Whether 1,3-dioxolane in the gastric fluid releases toxicologically relevant formaldehyde levels and whether these could reach the target organs is thus an open question, yet if that was the mechanistic concern, then a correct grouping would group all those acetals (cyclic or not) which are able to release formaldehyde under physiological conditions rather than grouping all cyclic acetals.

(C) Aromatic ethers^3^

This group is defined by “*an ether group—an oxygen atom connected to two aryl groups or one aryl and one alkyl group*”. This group encompasses 23 widely differing structures. Here, the key toxicological concern identified is for 4-methyl-anisole (EC 203-253-7) and methyl 2-naphthyl ether (EC 202-213-6), both self-classified as CMR2. In both cases developmental effects on different endpoints in presence of maternal toxicity were observed. Currently, we have no clear indication that an ether functionality next to an aryl group leads to an intrinsic toxicity and whether the toxicity of the two mentioned chemicals is specifically linked to the fact that they are aryl-ethers. Notably, 2-phenoxyethanol—which is not included in the group due to presence of an additional hydroxyl group—does contain the aromatic ether functionality and it is the cosmetic preservative with the probably strongest history of safe use.

(D) Aralkylaldehydes^4^

This group is defined by *the common presence of (a) aldehyde functionality and (b) a phenyl substituent which are linked by an alkyl chain*. The key toxicological concern in the group identified by ECHA is 2-(4-tert-butylbenzyl) propionaldehyde (EC 201-289-8), which has a harmonized classification as Repr.1B H360Fd. However, there is no indication in the literature that there is a toxicological property explained by the interaction of the aldehyde functionality and the phenyl ring in the molecules in this group—these are two independent chemical functions and thus it is scientifically questionable to base a grouping on the joint presence of these two groups in the same molecule. The reprotoxicity of 201-289-8 does not require the aldehyde group, as the corresponding acid (Lysmerylic acid; CAS 66735-04-4), the hydrocarbon *p-tert*-toluene (CAS 98-51-1) or the derived metabolite *p-tert-*butyl benzoic acid (CAS 98-73-7) have the same toxicity based on a common metabolite (Laue et al. 2017), completely independent of the aldehyde group but requiring the phenyl group, *inter alia*. On the other hand, the risk for skin sensitization mentioned as another toxicological endpoint in the ARN most likely comes from the aldehyde group—but then, a molecule with e.g. a cyclohexenyl instead of a phenyl ring would have similar sensitization potential based on our mechanistic understanding and would need to be included in the grouping. Thus, two functional groups defining the group in this ARN have independent toxicological importance and their common presence in a molecule does not justify a grouping, as no *similar toxicological properties* derive from their simultaneous presence in the same molecule.

(E) Paraben acid, salts and esters^5^

This group is defined by *the presence of the paraben acid moiety*, namely 4-hydroxy benzoic acid (EC 202-804-9) and includes both the acid, its salts and all its esters. This grouping would make perfect sense, if the concern would be based on the common metabolite, namely 4-hydroxy benzoic acid, as ample evidence shows that all paraben esters are rapidly hydrolyzed in vivo and that all administration routes lead to a single peak in plasma, corresponding to 4-hydroxybenzoic acid (Aubert et al. 2012).

However, the main toxicological concern in this group raised by ECHA are the esters, whereby Butyl paraben (EC 202-318-7) has been proposed as an endocrine disruptor (DK-EPA 2020), mainly based on its in vitro binding to the estrogen receptor, although at ≥ six orders of magnitude lower potency as compared to estrogen (Pop et al. 2018). In parallel Isobutyl Paraben (EC 224-208-8) was proposed as ED by Denmark. Both these assessments are not based on guideline-conform studies nor on ECHA registrations. The assessment of EC 202-318-7 has specifically excluded an assessment of metabolism, and the rapid conversion to 4-hydroxy benzoic acid was not considered in the assessment of EC 202-318-7. Interestingly, the ARN states that the ED concern for 4-hydroxy benzoic acid was refuted based on a Substance Evaluation conclusion document, which specifically stated that “*the estrogenic activity of 4-HBA is insignificant*” and that “*The concern in relation with endocrine disruption as main concern was not confirmed within this evaluation and 4-HBA is not regarded as having estrogenic activity*” (Czech_republic 2016).

Thus, the conclusions from this grouping is misleading, as it groups the acid with the esters and defines the acid as the common structural feature—which is well aligned with the known in vivo metabolism—but it maintains ED as the key concern of the group although an ECHA document has already concluded that there is no ED concern for the common metabolite actually defining the group. Based on this assessment, the acid should either not be grouped with the esters, or—by applying the sound toxicological grouping based on the common in vivo metabolite—this ARN would lead to a reassessment of the ED concern of the whole group, this time taking metabolism into account. Surprisingly, the grouping in the ARN does not lead to this scientific conclusion, it concludes that all substances—including the acid—should be evaluated for ED and all parabens potentially be restricted based on Substance of Very High Concern (SVHC) identification. It specifically states that “all the substances in the group are also suspected of having a reproductive toxicity hazard based on known or potential estrogenic mode of action”, despite the fact that methyl paraben (EC 202-785-7), the substance in the group produced at the highest tonnage, was registered with an OECD 443 extended one-generation reproductive toxicity (with F2 generation and developmental neurotoxicity) and a OECD Guideline 422 reproduction screening test, in both studies, no effects up to the maximal dose of 1000 mg/kg bw/d were observed (ECHA 2022b). Thus ECHA has the full set of data to conclude that there is no adverse effect and hence no ED identification on this key member of the group but did disregard this information when making these generalizations.

## Conclusion

In all these discussed groupings, there is no clear underlying toxicological mechanistic reason given why the common presence of the selected structural determinants should lead to a common toxicological outcome on the molecules in-cluded in the grouping. In none of these cases, the ANNEX XI requirement, namely that “*Substances whose (…) toxicological (…) properties are likely to be similar or follow a regular pattern as a result of structural similarity, may be considered as a group (…)”* appears to be fulfilled. Furthermore, grouping and common toxicological properties are in most cases specific to one or few toxicological endpoints. However, these groupings do not clearly delimit for which endpoints they should be applied. Most importantly, in none of the discussed ARN, a toxicological reason for the selection of the chemical functional group is given nor any toxicological considerations to delimit the boundaries of the group. These example show that these ARN follow a general principle applied by ECHA in recent ARN reports deviating from ANNEX XI and which is not justified by toxicological assumptions. ECHA writes that ARN are “*not part of the formal processes defined in the legislation but aims to support them*”—hence ARN are not legally binding, but still the ARN make conclusions and claims on the need for regulatory action, such as SVHC hazard classification, based on these grouping approaches. It also appears that the ARN conclusions are used to direct Member States to take action on individual substances e.g. in the preparation for REACH completeness checks (CCH) or harmonized hazard classification proposals (CLH). As an example, following the publication of the arylalkyl aldehydes ARN, Sweden promptly announced their intention for CLH proposals on all the substances that were judged in need of CMR 1b classifications in the ARN, see e.g., (ECHA 2022a). Other member states initiated at the same time completeness checks on other substances noted as requiring this assessment in the ARN. In the light of the upcoming importance of grouping under CSS—all grouping activities by ECHA should follow common, transparent, and scientifically defined toxicological principles. We call on ECHA to work with toxicology experts at large to develop a robust and aligned approach, that is based on sound science, and consistent with the guidance of Annex XI of the REACH Regulation. This will help build credibility in the future to produce robust grouping that can help accelerate ingredient assessments and leverage available data within the group. Such well-established grouping approaches had been published e.g., by the Research Institute for Fragrance Ingredients and associated scientists (Moustakas et al. 2022). The RIFM approach on safety assessments on well over 1000 fragrance substances in peer reviewed literature has shown clear benefit for applying grouping in managing the safe use of substances that are used in consumer products at low levels (< 0.01%). Also well-established principles of grouping utilized in the OECD Toolbox prescribed by ECHA for use by registrants, and the 12 grouping principles as recently published by BASF (Wohlleben et al. 2022) give a solid basis how scientific grouping can be done.

## Data Availability

All information on the individual chemicals and the ARN documents is freely available from the ECHA homepage at the respective links. This comment does not contain primary data.

## Footnotes

1 Published as GMT_208_cyclic_ethers_Report_public_20979_en.pdf.

2 Published as GMT_214_Cyclic_acetals_from_aldehydes_Report_public_21069_en.pdf.

3 Published as GMT_241_aromatic_ethers_Report_public_20845_en.pdf.

4 Published as GMT_305_Aralkylaldehydes_Report_public.pdf.

5 Published as GMT_310_Paraben_acid_salts_and_esters_Report_public_25989_en.pdf.
